# Preparation of Densified Fine-Grain High-Frequency MnZn Ferrite Using the Cold Sintering Process

**DOI:** 10.3390/ma16093454

**Published:** 2023-04-28

**Authors:** Yao Ying, Linghuo Hu, Zhaocheng Li, Jingwu Zheng, Jing Yu, Wangchang Li, Liang Qiao, Wei Cai, Juan Li, Daxin Bao, Shenglei Che

**Affiliations:** 1 College of Materials Science and Engineering, Zhejiang University of Technology, Hangzhou 310014, China; 2 Research Center of Magnetic and Electronic Materials, Zhejiang University of Technology, Hangzhou 310014, China; 3 Hengdian Group DMEGC Magnetics Co., Ltd., Dongyang 322118, China

**Keywords:** MnZn ferrite, cold sintering process, power loss, high frequency

## Abstract

The densified MnZn ferrite ceramics were prepared using the cold sintering process under pressure, with an acetate ethanol solution used as the transient solvent. The effects of the transient solvent, the pressure and annealing temperature on the density, and the micromorphology and magnetic properties of the sintered MnZn ferrites were studied. The densified MnZn ferrite was obtained using the cold sintering process and its relative density reached up to 85.4%. The transient solvent and high pressure are essential to the cold sintering process for MnZn ferrite. The annealing treatment is indispensable in obtaining the sample with the higher density. The relative density was further increased to 97.2% for the sample annealed at 950 °C for 6 h. The increase in the annealing temperature reduces the power loss at high frequencies.

## 1. Introduction

MnZn ferrites are the core materials in power transformers, sensors and other electronic devices due to their excellent soft magnetic performance, such as high initial permeability (*μ_i_*), high flux density (*B_s_*), low power loss (*P_cv_*) and relatively high Curie temperature (*T_C_*) [1,2,3,4,5,6]. With the continuous development of 5G communication technology and third generation wide band gap (WBG) semiconductors, electronic devices are developing toward miniaturization and high efficiency [7,8,9]. When the magnetic core in the electronic device is subjected to an AC magnetic field, some of the power is lost in the core and dissipated as heat and sometimes noise. The lost power is termed as power loss. Low power loss is the crucial factor for high efficiency in electronic devices. Increasing the operating frequency is the essential way to achieve miniaturization. However, *P_cv_* increases dramatically when the frequency increases to above 500 kHz, due to the dramatic increase in eddy current loss (*P*_ev_) and residual loss (*P*_rv_). In order to reduce the *P*_ev_ and *P*_rv_, it is necessary to refine the grain of the MnZn ferrite to nearly a single domain, namely smaller than 3 μm.

To achieve the purpose of grain refinement, an effective method is to reduce the sintering temperature [10,11]. In recent years, the cold sintering process (CSP), as an energy-efficient sintering technology, has emerged. The CSP is a technique according to which the densified ceramics or metals are sintered at low temperatures, typically below 300 °C and high pressure, with the aid of a transient solvent [12,13,14,15]. In the typical cold sintering process, the introduction of a certain amount of liquid is indispensable. The ceramic powder is uniformly wetted with a small amount of solvent, and the particle surface is decomposed and/or partially dissolved in the solvent and forms a supersaturated solution in the system. The application of high pressure at an appropriate temperature triggers the dissolved solute to precipitate in the interstices between the particles. This process is usually referred to as “dissolution precipitation” [12,16,17,18]. To date, some ceramics and metals with high density, ranging from mono-, di-, ternary, quaternary and pentameric compounds, have been successfully prepared using CSP [19,20]. Zinc ferrite was successfully prepared using CSP and the sample density reached 93% of the theoretical density after annealing treatment [21]. Mingming Si [22] prepared zinc oxide/polyetheretherketone (PEEK) composites with excellent performance using the cold sintering method.

In order to refine the grain of MnZn ferrite to nearly a single domain with a grain size of less than 3 μm, the cold sintering process was employed in this work. The cold sintering process can inhibit the growth of grains during the ceramic sintering process and obtain a densified ceramic with uniform and fine-grain size. The cold sintering process can effectively reduce the volatilization of the elements, which occurs in the traditional sintering process due to the high sintering temperature. To focus on the effect of the cold sintering process during the preparation of the fine-grain MnZn ferrite, grain boundary additives were not added to this series of samples. The effects of the transient solvent, the pressure and annealing temperature on the density, and the micromorphology and magnetic properties of the sintered MnZn ferrite were studied. The densified MnZn ferrite was successfully prepared. The relative density of the as-CSPed sample reached up to 85.4%. After annealed at 950 °C for 6 h, the relative density of the sample was further increased to 97.2%.

## 2. Experimental

**Figure 1:**
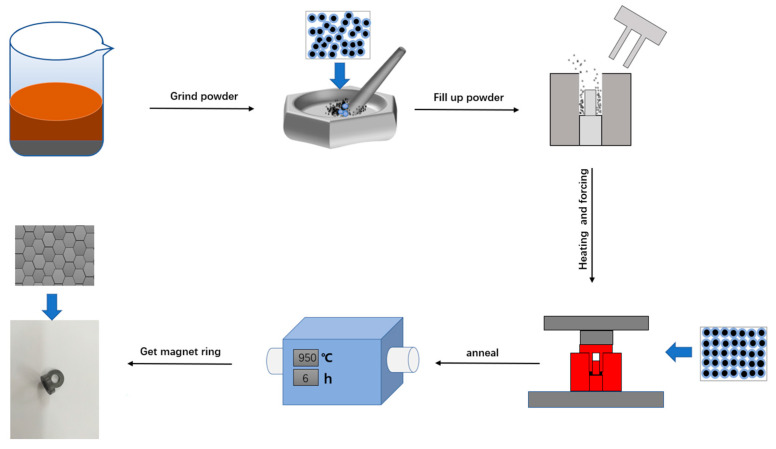
Flow chart of the cold sintering process for MnZn ferrite.

MnZn ferrite powders were firstly prepared by solid state reaction. The raw materials, namely Fe_2_O_3_, Mn_3_O_4_ and ZnO, with a purity of 99% were weighed, according to the stoichiometry of Mn_0.888_Zn_0.049_Fe_2.063_O_4_. This composition was also used to investigate the effect of low-temperature sintering on MnZn ferrites in our previous work [11]. It was beneficial for us to compare the different properties of the MnZn ferrites sintered by these two processes. The mixtures were calcined at 970 °C in the nitrogen atmosphere. After that, the obtained MnZn ferrite was ball milled into powders. The flow chart of the CSP for MnZn ferrite is shown in Figure 1. Firstly, the calcined MnZn ferrite powder (50 g) and ethanol solution acetic acid (150 g) with a concentration of 4 M were mixed in a beaker and then stirred for 24 h. After pretreatment, the beaker was placed in an oven at 80 °C for 6 h to obtain the dried powder. Then 12 wt% acetate ethanol at a concentration of 2 M was added to the dried powder and they were ground together in a mortar for 30 min. The cold sintering process was carried out in a steel mold with an outer diameter of 16 mm and an inner diameter of 8 mm, which was surrounded by a heating element with a temperature control system. Then, 2 g mixtures were placed in the steel mold and heated to 330 °C at a rate of 4 °C/min. Meanwhile, uniaxial pressure was applied to the mold vertically for 2 h. Finally, the samples obtained by cold sintering were annealed for 6 h at higher temperatures (850 or 950 °C) in an atmosphere of nitrogen.

The crystal structures of the samples were examined from 10 to 80° with a scanning speed of 20°/min using an X-ray diffractometer (XRD) (Ultima IV, Rigaku, Japan) with Cu *K_α_* radiation. The microstructure and cross-sectional morphology of the sintered samples were observed using a scanning electron microscope (SEM) (SU1510, Hitachi, Japan). Prior to the SEM measurements, the cross-sections were sanded using sandpaper of different sizes, from 400 to 7000 mesh, and then etched in 4% HF acid solution for 90 s. The average grain size (*D*) was estimated. The infrared (IR) spectra were measured using an infrared spectrometer (Nicolet 6700, Thermo, USA). The thermal gravimetric (TG) curves were measured in the temperature range from 25 to 900 °C using a thermal gravimetric analyzer (TG209 F1, Netzsch, Germany). The sample density (*ρ*) was measured using the Archimedes drainage method. The *P_cv_* was measured using an AC BH curve analyzer (SY-8218, IWATSU, Japan).

## 3. Results and Discussion

### 3.1. Effect of Solvents on the Cold Sintering

**Figure 2:**
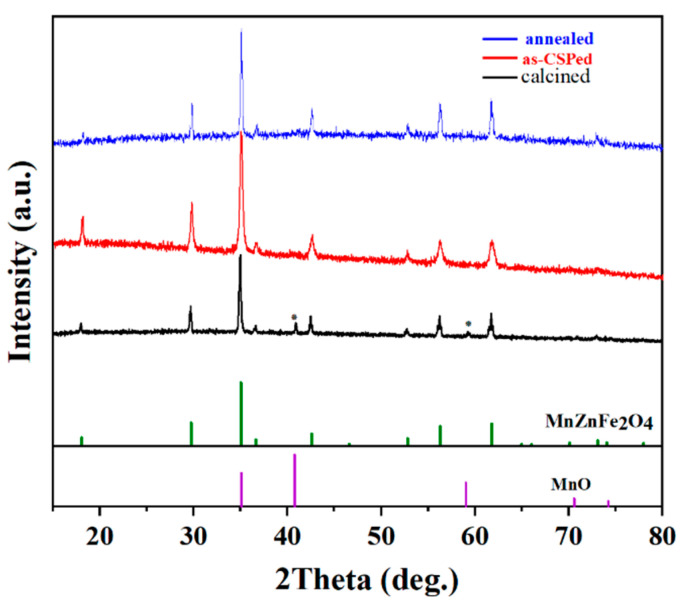
XRD of the calcined, as-CSPed and annealed MnZn ferrite samples.

Figure 2 shows the XRD patterns of the calcined, as-CSPed and annealed MnZn ferrite samples. The diffraction peaks of the calcined sample are basically consistent with those of the MnZn ferrite, but two impure diffraction peaks of MnO were found at around 41.1° and 60°. The MnO peaks disappeared in the as-CSPed and annealed samples. During the calcination process, the raw materials of Fe_2_O_3_, Mn_3_O_4_ and ZnO undergo a solid-state reaction and, consequently, form spinel phase MnZn ferrite with releasing oxygen. The equilibrium oxygen partial pressure of the solid-state reaction is higher at a high temperature. In this work, the calcination was carried out in nitrogen, so the reaction atmosphere was reductive compared to the equilibrium oxygen partial pressure. Therefore, Mn_3_O_4_ partially reverted to MnO during the calcination. It was also found that the diffraction peaks of the as-CSPed sample were wider than those of the calcined sample. In CSP, the acetic acid treatment leads to the formation of an amorphous layer on the surface of the powder, which weakens the crystallinity of the powder. Therefore, the XRD peaks become wider.

**Figure 3:**
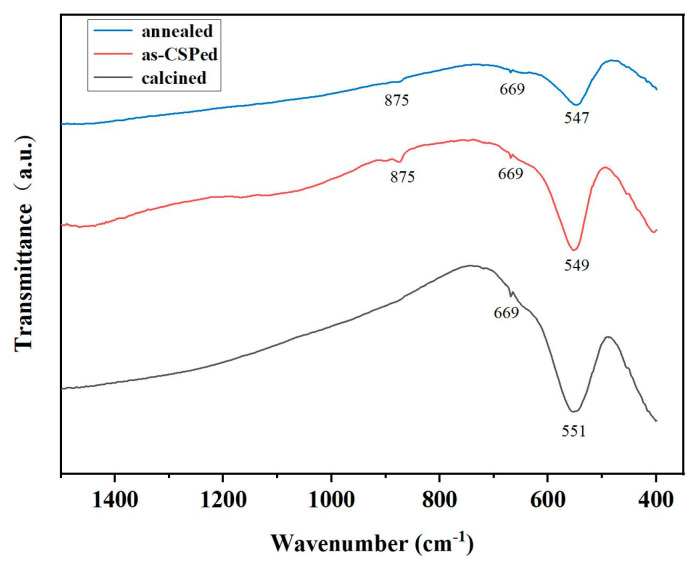
Infrared spectra taken in the frequency range 400–1500 cm^−1^ for the calcined, as-CSPed and annealed MnZn ferrite samples.

To detect the valency change of the Mn ions, the infrared (IR) spectra were measured for the calcined, as-CSPed and annealed MnZn ferrite samples, as shown in Figure 3. It was found that three absorption peaks appeared at 549, 669 and 875 cm^−1^. These peaks hardly changed for the calcined, as-CSPed and annealed MnZn ferrite samples. The absorption peak for the Mn^2+^ ion in the manganese ferrite ranged from 545–550 cm^−1^ [23]. Therefore, the 549 cm^−1^ peak in this series of samples was ascribed to Mn^2+^ ion. In Mn_2_O_3_, the characteristic absorption peaks appeared at 668 and 870 cm^−1^ [24]. Therefore, the 669 and 875 cm^−1^ peaks were ascribed to Mn^3+^ ion. It is obvious that the 669 and 875 cm^−1^ peaks were very weak and the 549 cm^−1^ peak was much stronger than these two peaks in this series of samples. Thus, Mn ions in this series of samples mainly existed in the form of Mn^2+^, and the number of Mn^3+^ was very small.

Since MnZn ferrite is partially soluble in acetic acid, an ethanol solution of acetate was chosen as the transient solvent for the cold sintering process [21]. During the cold sintering process, Mn, Zn and Fe dissolve in the transient solvent in the ionic state. With an increase in pressure at high temperature, MnZn ferrite grains begin to contact each other and form necks at the contact points, and Mn^2+^, Zn^2+^ and Fe^3+^ ions also gradually diffuse to the vicinity of the grain neck with the solvent and precipitate. Thus, the diffraction peaks for MnO disappear in the XRD pattern of the as-CSPed sample. The diffraction peaks of the annealed sample are narrower than those of the as-CSPed sample, as shown in Figure 2. It may be due to the fact that the gain size of the annealed samples becomes larger.

**Figure 4:**
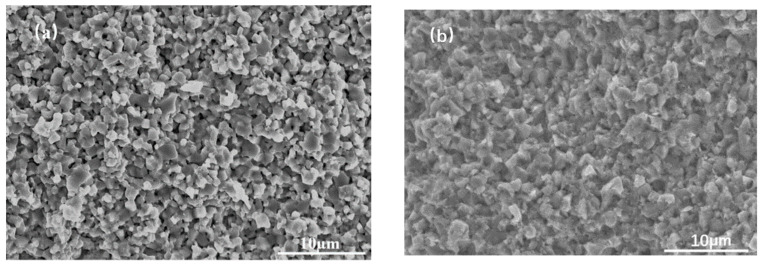
Fractural SEM images of the sample after cold sintering: (**a**) without ethanol acetate solution and (**b**) with 12 wt% ethanol acetate solution.

Figure 4 shows the SEM fractural morphology of the samples without the ethanol solution of acetate and with the 12 wt% ethanol solution of acetate after the cold sintering process. It can be seen that the grain adhesion without the ethanol solution of acetate is poor and there are many pores. The sintering traces are not obvious. The sample with the ethanol solution of acetate, as a transient solvent, has better particle adhesion and less pores, exhibiting better sintering. By measuring the sample density, the density of the as-CSPed sample without the ethanol solution of acetate was 3.53 g/cm^3^, while that with the ethanol solution of acetate increased to 4.08 g/cm^3^. Thus, the introduction of the moderate ethanol solution of acetate is beneficial for increasing the densification in the CSP.

The experimental setup can be regarded as a well-closed system. Due to the slow heating rate, the transient solvent can be retained for a long time. Therefore, the system is in a non-equilibrium state before the solvent completely evaporates. The acetic acid solution has a wetting effect on the grains, which reduces the friction between grains. At the same time, the Mn^2+^, Zn^2+^ and Fe^2+^ ions on the grain surface dissolve, and defects are formed in the MnZn ferrite grain surface. Under the action of high stress, the local pressure between the two grains is very high, which enhances the dissolution of the contact interface between the solid and the liquid. Due to the greater chemical potential in the contact area between the particles, the particles diffuse through the liquid phase and precipitate at locations away from the force contact area. During the holding process, the solid transfer accelerates, the supersaturation increases significantly as the transient solvent evaporates, and small particles begin to grow, which leads to larger grains [25,26].

### 3.2. Effect of Pressure on Cold Sintering

**Figure 5:**
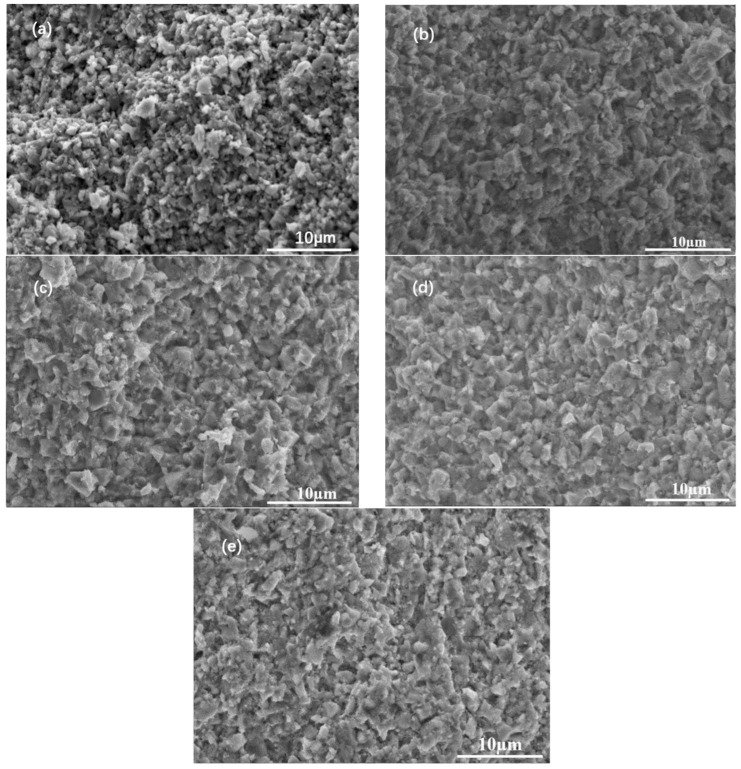
Morphology of the MnZn ferrites after cold sintering under the pressure: (**a**) 600, (**b**) 800, (**c**) 1000, (**d**) 1200 and (**e**) 1400 MPa at 330 °C for 2 h.

Figure 5 shows the fractural morphology of the sample after the cold sintering under different pressures. When the pressure is lower than 1000 MPa, most of the grains exist individually and the pores are obvious. As the pressure exceeds 1000 MPa, some grains connect, and the pores between the grains become smaller. This indicates that high pressure promotes the cold sintering.

In the conventional sintering process, the driving force for powder consolidation is provided by thermal energy at the high sintering temperature. In the cold sintering process, the driving force is mainly provided by the mechanical energy originating from the large external pressure, and the applied pressure increases the solubility of the particles with sharp edges. In this system, a relatively high temperature of 330 °C and a high pressure exceeding 1000 MPa promotes the solubility of the MnZn ferrite and, meanwhile, the evaporation of the liquid phase, which results in the formation of a supersaturated liquid and mass transport via diffusion.

Due to the synergy of the external applied pressure and the capillary stress exerted by the liquid, the atoms diffuse through the liquid from the higher chemical potential areas to the regions of lower chemical potential, where precipitation occurs [18]. The contact areas are found at high chemical potential, and precipitation occurs instead where the chemical potential is lower, away from the contact areas. This is demonstrated by the morphological change of the sample, as shown in Figure 5. As the pressure increases, the original pores between the grains are filled with substances, linking the grains and the grains become tighter.

**Figure 6:**
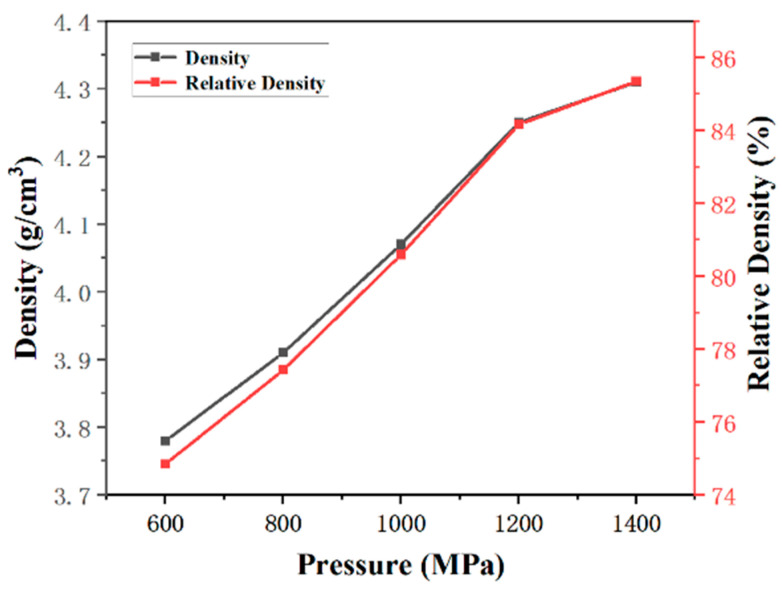
Densities and relative densities of the samples after cold sintering under different pressures.

Figure 6 shows the densities and relative density of the samples after cold sintering at different pressures. The density of sample increases with the increasing pressure and reaches 4.31 g/cm^3^. The relative density reaches 85.4%. This also implies that the applied pressure improves the CSP densification.

A sintering body with a relative density of over 85% can be considered to be almost dense enough in some cases of usage, but it is still lower than the normal relative density of ferrite cores, i.e., 90%. Subsequently, an annealing treatment was investigated.

### 3.3. Effect of Annealing Treatment

**Figure 7:**
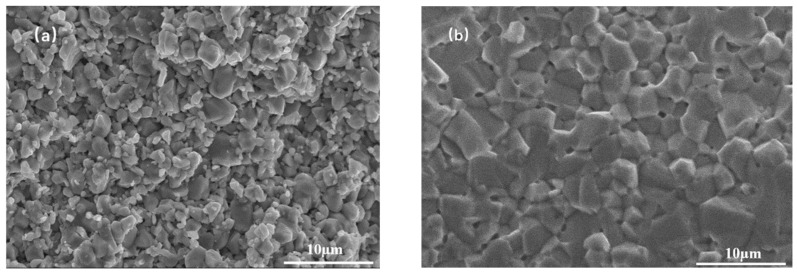
SEM images of the MnZn ferrite samples after annealing at: (**a**) 850 °C and (**b**) 950 °C for 6 h.

A fully densified structure may not be achieved for the MnZn ferrite using just a one-step CSP, due to the limited dissolution and formation of the grains. In this case, a post-annealing process is necessary to further increase the crystallinity and the density. Figure 7 shows the fractural morphology of the MnZn ferrite samples after annealing at high temperatures of 850 and 950 °C for 6 h. After being annealed at 850 °C, the sample is not dense and many pores exist, which means that 850 °C is not high enough for densification. As the annealing temperature increases to 950 °C, the grains grow, the pores decrease and the sample exhibits good densification.

**Figure 8:**
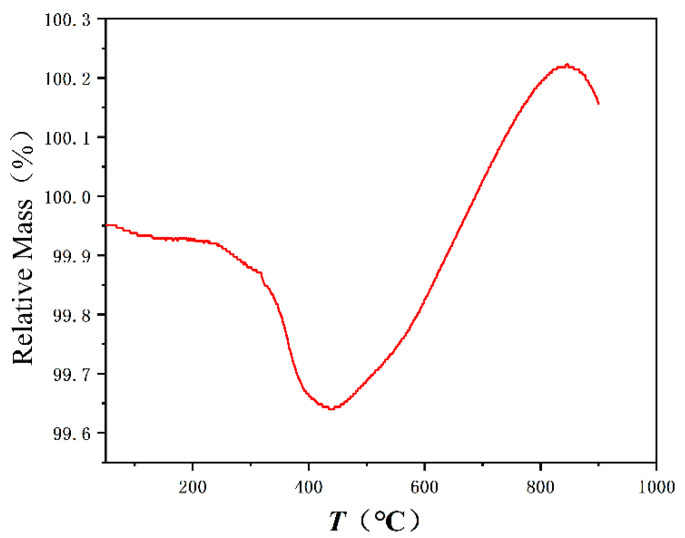
TG curve of the as-CSPed sample.

We further measured the TG curve of the as-CSP sample (Figure 8). When the temperature exceeds 850 °C, the TG curve starts to decrease. This indicates the further solid-phase reaction and the densification process. When the sample is annealed at 850 °C, the solid-phase reaction between the powders is not sufficient, there still exist many pores in the sample and the degree of densification is not enough. When increasing the annealed temperature, the solid-phase reaction is promoted, grains are grown and the pores are removed, so the density increases.

**Figure 9:**
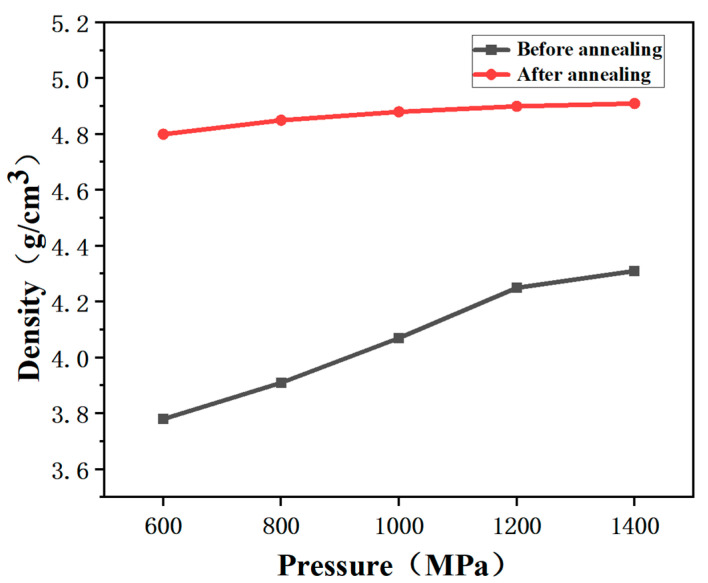
Densities of the samples with different CSP pressures after annealing at 950 °C.

Figure 9 shows the CSP pressure dependence of the density of the samples before and after annealing at 950 °C for 6 h. The density of the samples with different pressures before annealing was 3.78–4.31 g/cm^3^, and the density increased to 4.80–4.91 g/cm^3^ after annealing at 950 °C.

**Figure 10:**
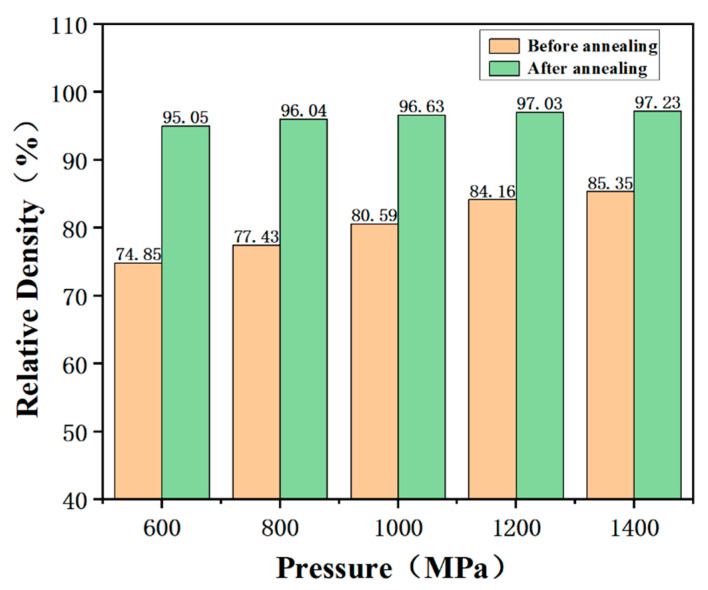
Relative densities of the samples with different CSP pressures after annealing at 950 °C.

Therefore, the annealing treatment significantly enhanced the density of the sample. Figure 10 shows the relative densities of the samples with different CSP pressures after annealing. The relative density increased remarkably after annealing. The highest relative density increased up to 97.2%, which is compared with the densities of most MnZn ferrites prepared using the conventional high-temperature sintering process. The conventional high-temperature sintering process requires the use of temperatures above 1100 °C to densify the MnZn ferrite.

**Figure 11:**
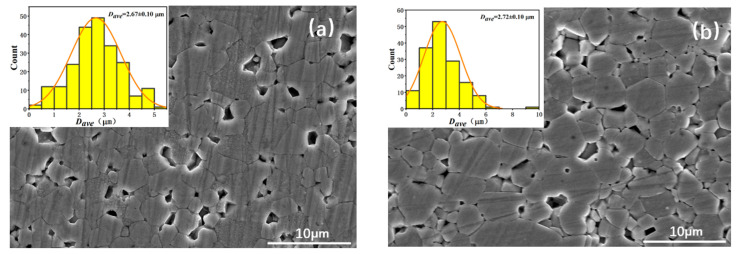

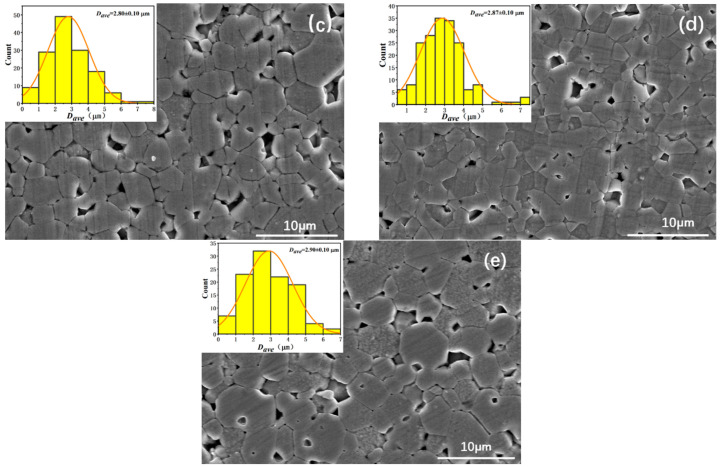
SEM images of samples with different CSP pressure: (**a**) 600, (**b**) 800, (**c**) 1000, (**d**) 1200 and (**e**) 1400 Mpa after annealing at 950 °C.

After the treatment of grinding and etching, the fractural images of the samples at different pressures after annealing at 950 °C were measured by SEM. Figure 11 shows the corresponding SEM images of the samples. The statistical histogram of the particle size is shown in the corresponding illustration. The grain size was calculated and is shown in Table 1. For the sample with the CSP pressure below 1000 MPa, there are some pores between the grains and the sample is not very compact. When the pressure increases up to above 1200 MPa, the pores decrease and the sample becomes more compact. Therefore, the increase in pressure promotes densification of the sample after annealing. At the same time, the average grain size also increases from 2.67 to 2.90 μm. This is attributed to the fact that the pressure during the cold sintering stage promotes the growth of crystal grains. Due to the no grain boundary phase formed in these samples, there also exist some pores caused by the small grain abscission in the etching process. The average grain size in this series of samples was less than 3 μm, which was also less that the critical size (3.8 ± 0.7 μm) of a single domain in MnZn ferrites. Reducing the grain size below the single-domain size is beneficial to reduce the power loss at high frequencies [27].

### 3.4. Magnetic Properties

**Figure 12:**
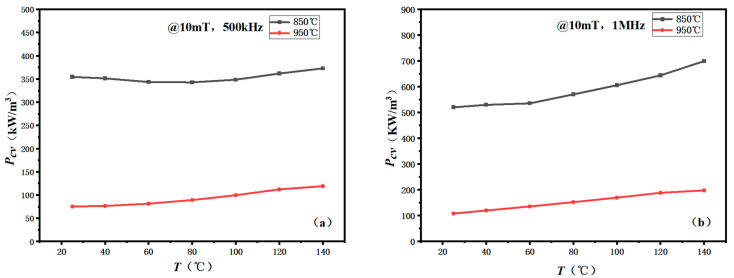
The *P_cv_* (*T*) curves of the CSP samples at different frequencies: (**a**) 500 kHz and (**b**) 1 MHz after annealing for 6 h.

**Figure 13:**
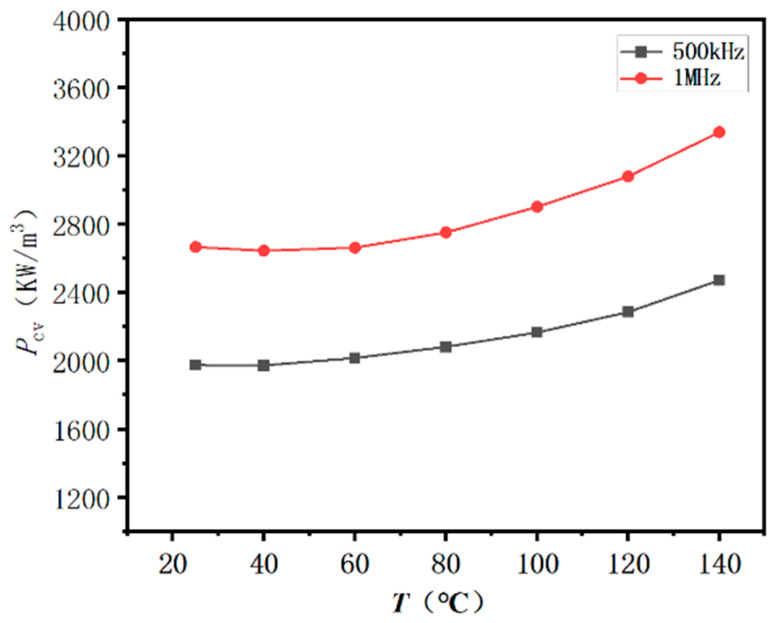
Temperature dependence of *P_cv_* for the sample annealed at 950 °C under 500 kHz/30 mT and 1 MHz/30 mT.

Figure 12 shows the temperature dependence of the *P_cv_* [*P_cv_*(*T*)] for the samples after annealing at 850 and 950 °C for 6 h, under the conditions of 500 kHz/10 mT and 1 MHz/10 mT. The power loss of MnZn ferrite increases with temperature and frequency. The *P_cv_* of MnZn ferrite annealed at 850 °C was 342 and 520 kW/cm^3^ at 500 kHz/10 mT and 1 MHz/10 mT, 25 °C, respectively. When the annealing temperature increased to 950 °C, the *P_cv_* was greatly reduced to 75 and 108 kW/m^3^ at 500 kHz/10 mT and 1 MHz/10 mT, respectively. Figure 13 shows the *P_cv_*(*T*) curves for the annealed sample after annealing at 950 °C for 6 h, under the conditions of 500 kHz/30 mT and 1 MHz/30 mT. The *P_cv_*(*T*) curves show similar behavior to those under 10 mT. When the magnetic flux density increased from 10 to 30 mT, the *P_cv_* increased.

As the annealing temperature increases, the density of the sample increases and the porosity decreases. Thus, the power loss decreases. However, compared to the traditional sintering process, the power loss of the CSP samples was higher. The grain boundary of MnZn ferrites can only be formed above 1000 °C [28]. In the conventional sintering process, additives such as CaCO_3_, SiO_2_ and V_2_O_5_ are generally added to form a high-resistance glass phase on the grain boundary [29,30,31,32,33]. The formation of the high-resistance grain boundary will remarkably reduce the *P_cv_*. In this series of samples, the high-resistance grain boundary was not formed. In the next work, the grain boundary additives and sintering aid will be co-added to improve the formation of the high-resistance grain boundary and to reduce the power loss. Through the construction of the high-resistance grain boundary in the fine-grained MnZn ferrite, the power loss at a high frequency may be reduced dramatically and make it a good candidate material with a magnetic core for the high-frequency switching power supply.

## 4. Conclusions

In this study, MnZn ferrites with fine grains and high density were prepared using the cold sintering process. It is found that the transient solvent and high pressure are the key factors for the CSP process for MnZn ferrite. The as-CSPed MnZn ferrite with a relative density of up to 85% was obtained. After being annealed at 950 °C for 6 h, the relative density was further increased to 97%, while the average grain size was maintained at less than 3.0 μm. The increase in the annealing temperature reduces the *P_cv_*s at high frequencies of 500 kHz and 1 MHz.

This work confirms that it is feasible to prepare densified fine-grain MnZn ferrite using CSP, which is a different process from the traditional low-temperature sintering process. The grains of the sample prepared by CSP are more uniform and the average grain size was smaller than the critical size of a single domain. The average grain size in this series of samples was also smaller than that in the sample prepared by the low-temperature sintering process [11]. This work lays a solid foundation for the follow-up development of low-*P_cv_* MnZn ferrite by CSP for high frequency applications.

## Figures and Tables

**Table 1:** Average grain size of the MnZn ferrites sintered at different CSP pressures.

Pressure (MPa)	600	800	1000	1200	1400
*D_ave_* (µm)	2.67	2.72	2.80	2.87	2.90

## Data Availability

The data presented in this study are available on request from the corresponding author. The data are not publicly available due to privacy.
